# Sodium lauryl ether sulfate (SLES) degradation by nitrate-reducing bacteria

**DOI:** 10.1007/s00253-017-8212-x

**Published:** 2017-03-15

**Authors:** Ana M. S. Paulo, Rozelin Aydin, Mauricio R. Dimitrov, Harm Vreeling, Ana J. Cavaleiro, Pedro A. García-Encina, Alfons J. M. Stams, Caroline M. Plugge

**Affiliations:** 1 Laboratory of Microbiology, Wageningen University, Stippeneng 4, 6708 WE Wageningen, The Netherlands; 2 Department of Chemical Engineering and Environmental Technology, University of Valladolid, Calle Dr. Mergelina s/n, 47011 Valladolid, Spain; 3 Centre of Biological Engineering, University of Minho, 4710-057 Braga, Portugal; 4 Department of Bioengineering, Adana Science and Technology University, 01180 Seyhan/Adana, Turkey; 5 Department of Microbial Ecology, Netherlands Institute of Ecology (NIOO-KNAW), Droevendaalsesteeg 10, 6708 PB Wageningen, The Netherlands

**Keywords:** Anionic surfactants, Denitrification, *Pseudomonas*, Sodium lauryl ether sulfate

## Abstract

The surfactant sodium lauryl ether sulfate (SLES) is widely used in the composition of detergents and frequently ends up in wastewater treatment plants (WWTPs). While aerobic SLES degradation is well studied, little is known about the fate of this compound in anoxic environments, such as denitrification tanks of WWTPs, nor about the bacteria involved in the anoxic biodegradation. Here, we used SLES as sole carbon and energy source, at concentrations ranging from 50 to 1000 mg L^−1^, to enrich and isolate nitrate-reducing bacteria from activated sludge of a WWTP with the anaerobic-anoxic-oxic (A^2^/O) concept. In the 50 mg L^−1^ enrichment, *Comamonas* (50%), *Pseudomonas* (24%), and *Alicycliphilus* (12%) were present at higher relative abundance, while *Pseudomonas* (53%) became dominant in the 1000 mg L^−1^ enrichment. *Aeromonas hydrophila* strain S7, *Pseudomonas stutzeri* strain S8, and *Pseudomonas nitroreducens* strain S11 were isolated from the enriched cultures. Under denitrifying conditions, strains S8 and S11 degraded 500 mg L^−1^ SLES in less than 1 day, while strain S7 required more than 6 days. Strains S8 and S11 also showed a remarkable resistance to SLES, being able to grow and reduce nitrate with SLES concentrations up to 40 g L^−1^. Strain S11 turned out to be the best anoxic SLES degrader, degrading up to 41% of 500 mg L^−1^. The comparison between SLES anoxic and oxic degradation by strain S11 revealed differences in SLES cleavage, degradation, and sulfate accumulation; both ester and ether cleavage were probably employed in SLES anoxic degradation by strain S11.

**Electronic supplementary material:**

The online version of this article (doi:10.1007/s00253-017-8212-x) contains supplementary material, which is available to authorized users.

## Introduction

Anionic surfactants account for 60% of worldwide surfactants production (Holmberg et al. 2002) and sodium lauryl ether sulfate (SLES) is one of the most commonly used. SLES is a mixture of linear primary alkyl ether sulfates (AES) present in the formulation of several commercial detergents and personal care products (Khleifat 2006). The average concentration of anionic surfactants in domestic wastewater can vary between 0.4 and 12 mg L^−1^ (HERA 2002; HERA 2004; HERA 2013), although higher concentrations are frequently present in industrial wastewater, e.g., from the cosmetic industry, or in wastewater from surfactant-based technologies used for the cleanup of contaminated soils and aquifers (Huang et al. 2015; Shah et al. 2016; Zhang et al. 1999). The concentrations used in these processes are generally close or higher than the surfactant critical micelle concentration (CMC) (Ruckenstein and Nagarajan 1975). CMC corresponds to a minimum in the surface tension value, and thus solubilization of hydrophobic compounds is better achieved at concentrations higher than the CMC (Haigh 1996). For example, SLES forms micelles at a concentration higher than 300 mg L^−1^ (Aoudia et al. 2009), and 3000 mg L^−1^ was the concentration of anionic surfactants (mainly SLES) in the wastewater from a cosmetic production plant (Aloui et al. 2009).

Wastewater with high concentration of surfactants may deteriorate the biological treatment in wastewater treatment plants (WWTPs), e.g., by causing a decrease in floc size of activated sludge and/or by creating excessive foam in aerated compartments (Liwarska-Bizukojc and Bizukojc 2006; Wagener and Schink 1987). When applied at concentrations above the CMC, many surfactants become toxic to microorganisms by binding to enzymes, structural proteins, and phospholipids or by changing the hydrophobicity of the bacterial cell (Cserháti et al. 2002; Willumsen et al. 1998).

**Fig. 1:**
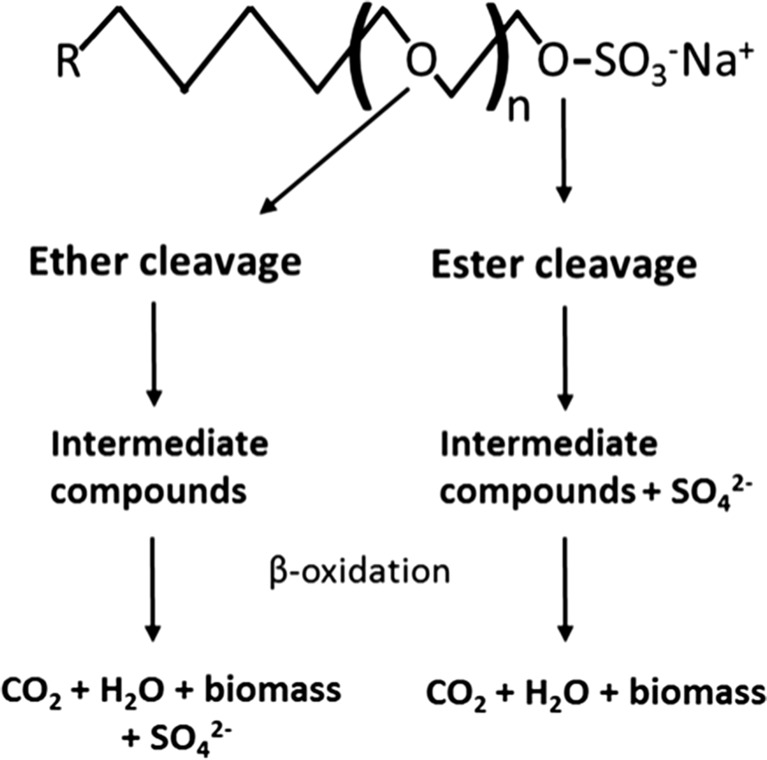
Scheme of possible SLES cleavage mechanisms for complete degradation to CO_2_ and biomass formation. The general molecular structure of SLES is shown, where *n* is the mean of ethoxy units (*n* = 2–3 in commercial products), and *R* is the alkyl group (the linear alkyl chain of AES surfactants can have 12 to 18 carbons) (adapted from Hales et al. (1986) and Steber and Berger (1995))

SLES can be degraded by different aerobic bacteria, namely *Citrobacter braakii* and a consortium of *Acinetobacter calcoacetiacus*, *Klebsiella oxytoca*, and *Serratia odorifera* (Dhouib et al. 2003; Khleifat 2006; Swisher 1987). Aerobic degradation of linear primary AES occurs mainly by ether cleavage (Budnik et al. 2016; Hales et al. 1986; Steber and Berger 1995; White et al. 1996), with the formation of intermediate compounds which can be further degraded and release sulfate (Fig. 1). Another possible mechanism is ester cleavage of the AES (Fig. 1), by which sulfate is directly split of, before the degradation of the carbon body (Hales et al. 1986). Thus, the presence of sulfate can be used as an indication of SLES cleavage and degradation.

Facultative anaerobic bacteria are present in the anoxic (nitrate-reducing) and oxic compartments of WWTPs (Gerardi 2002) and might play an important role in surfactant degradation. In a WWTP with the anaerobic-anoxic-oxic (A^2^/O) concept, surfactants are possibly degraded in the anoxic compartment. However, nothing is known about AES or SLES degradation at anoxic (nitrate-reducing) conditions. In this study, SLES concentrations below and above the CMC value were used to enrich and isolate nitrate-reducing bacteria from activated sludge. The bacterial community structure of the anoxic enrichments was studied. Several isolates were obtained and three isolates that degrade SLES with nitrate were compared. A *Pseudomonas nitroreducens* strain turned out to be the best SLES degrader.

## Materials and methods

### Enrichment of SLES-degrading bacteria

Activated sludge from a WWTP (Valladolid, Spain) with the A^2^/O concept was used as inoculum. Enrichments were performed in batch 120-mL serum bottles containing 40 mL of medium. The anoxic medium was prepared under dinitrogen gas, and the bottles were flushed several times with dinitrogen gas (1.5 × 10^5^ Pa final pressure). Bottles were sealed with butyl-rubber stoppers and crimp seals. The mineral salts medium contained (per liter demineralized water): 1 g KH_2_PO_4_, 3.48 g Na_2_HPO_4_·2H_2_O, 1 g (NH_4_)_2_SO_4_, 0.033 g MgCl_2_·6H_2_O, 0.0090 g CaCl_2_·2H_2_O, and 0.01 g Fe(NH_4_) citrate. Vitamins and trace elements were as described by Holliger et al. (1993). SLES was used as sole carbon and energy source and added from a filter-sterilized anoxic stock solution. Commercial SLES (information given by the manufacturer: *M*_*w*_ = 385 g mol^−1^; average of 2 degrees of ethoxylation (*n* = 2; Fig. 1); 70% active; alkyl chain with an average of 12 carbons) with the commercial name Marlinat 242/70 was purchased from Sasol (Hamburg, Germany). SLES concentrations tested were 50, 100, 250, 500, and 1000 mg SLES L^−1^, below and above the CMC of SLES (300 mg L^−1^) (Aoudia et al. 2009). SLES concentrations in mg L^−1^ or mmol L^−1^ were calculated considering the content of SLES (70%) in the commercial compound and the molecular weight indicated above. KNO_3_ was added as electron acceptor from a sterilized anoxic stock solution to a final concentration of 10 mmol L^−1^. Unless otherwise stated, batches were incubated statically at 30 °C, and the pH was 7.3 ± 0.1. Following bacterial growth and absence of foam formation (visual inspection after stirring the bottle), 10% (*v*/*v*) of the culture was transferred eight times to fresh medium, always containing the same SLES concentration. After 1 week of incubation of the eighth transfer, nitrate was analyzed for all the enrichments.

### Isolation and identification

Dilutions of the anoxic enriched cultures with 50, 250, and 1000 mg SLES L^−1^ were streaked on agar plates containing tryptic soy broth and 20 g L^−1^ of agar noble (BD Difco, Franklin Lakes, NJ). The plates were incubated aerobically at 30 °C. Colonies with different morphology were selected and streaked on new plates until pure cultures were obtained. For identification, cells of each isolate were picked from single colonies, diluted in 10 μL of sterilized DNA-free distilled water and lysed for 10 min at 95 °C. Lysates were stored at −20 °C. The 16S rRNA genes of lysates were amplified by PCR as described by van Gelder et al. (2012). BLASTN was used for identifying closely related 16S rRNA gene sequences and BLASTN alignment tool was used to compare the 16S rRNA sequences of the obtained isolates (Altschul et al. 1990).

The 16S rRNA gene sequences of strains S7, S8, and S11 have been deposited in the GenBank database under accession numbers KJ152584, KJ152585, and KJ152586, respectively. Strains S7, S8, and S11 and have been deposited in DSMZ (German Collection of Microorganisms and Cell Cultures, Braunschweig, Germany) with the accession numbers DSM 28624, DSM 28647, and DSM 28648, respectively.

### Bacterial community profiling of the enrichments

#### Denaturing gradient gel electrophoresis (DGGE)

DGGE was used to compare the bacterial communities of all the anoxic enrichments developed with different SLES concentrations, as well as the 16S rRNA amplicons of selected isolates. Approximately 40 mL aliquots of well-homogenized enriched cultures were concentrated by centrifugation (10,000*g*, 10 min) and immediately stored at −20 °C. Genomic DNA from enriched cultures was extracted with the FastDNA® Spin kit for soil (MP Biomedicals, Santa Ana, CA) according to the manufacturer’s protocol. PCR of partial bacterial 16S rRNA genes was performed as described in Alves et al. (2013). After the completion of the electrophoresis, gels were silver-stained (Sanguinetti et al. 1994) and scanned.

#### 454 Pyrosequencing analysis

After DGGE profile analysis, three enrichment cultures were selected (50, 250, and 1000 mg SLES L^−1^ enrichments) for bacterial diversity analysis and identification by 454 pyrosequencing. Sample preparation, DNA sequencing and data processing were performed according to Dimitrov et al. (2014). Samples were rarefied to an equal number of sequences (12,310 sequences). Alpha diversity metrics (Chao1 and Shannon indexes) were calculated using alpha_rarefaction.py workflow script available in QIIME (http://qiime.org/scripts/alpha_rarefaction.html). In order to cross-check the taxonomical classification obtained by the QIIME pipeline for the most abundant operational taxonomic units (OTUs), a selection of representative OTUs was matched with the GenBank nucleotide database using BLASTN (http://ncbi.nlm.nih.gov/blast). Only results with at least 98% maximum similarity were considered. The raw sequence data obtained for the three enrichment cultures were deposited in Sequence Read Archive (SRA) from NCBI database, under the accession number SRP077858, associated to the BioProject with accession number PRJNA326920.

### Growth and degradation tests

The ability of seven isolates to degrade SLES under anoxic conditions was tested by transferring a single colony back to batch liquid cultures, using a SLES concentration of 100 mg L^−1^ and KNO_3_ (10 mmol L^−1^) as electron acceptor. Based on these incubations and 16S rRNA gene comparison results, three strains (S7, S8, and S11) were selected for growth and degradation studies.

#### SLES degradation

Growth and SLES degradation by strains S7, S8, and S11 were tested with SLES as sole carbon and energy source at a final concentration of 500 mg L^−1^ (1.3 mmol L^−1^). For this, triplicate 250-mL serum bottles with 80 mL of medium were prepared as described for enrichments, but a sulfate-free mineral salts medium was used, to determine sulfate release from SLES. The sulfate-free medium contained (per liter demineralized water) 0.81 g NH_4_Cl instead of 1 g (NH_4_)_2_SO_4._ KNO_3_ was added to a final concentration of 30 mmol L^−1^, due to the higher SLES concentration used in these assays. Each bottle was inoculated with 2% (*v*/*v*) of an active bacterial culture grown with SLES and nitrate. Optical density (OD) measurements at 600 nm were performed and nitrate, nitrite, and sulfate were analyzed in time, until the stationary growth phase was reached. SLES concentration was measured at the beginning and end of each incubation using a kit for anionic surfactant quantification. This measurement was also used for determining SLES cleavage. Dissolved organic carbon (DOC) measurements were performed at the beginning and end of the assay to determine SLES conversion to biomass and CO_2_.

Duplicate control batch tests without SLES were always included, to confirm that SLES was the only carbon and energy source present in the medium, as well as duplicate controls without nitrate, to confirm that nitrate was the only electron acceptor used by bacteria.

#### Effect of increased concentrations of surfactants

Strains S8 and S11 were further compared for their ability to grow and reduce nitrate in the presence higher SLES concentrations (1, 5, 10, and 20 g L^−1^). The ability of these two strains to reduce nitrate (20 mM) with 40 g L^−1^ SLES was also checked. Anoxic growth and degradation with sodium dodecyl sulfate (SDS) (1 and 10 g SDS L^−1^) by strains S8 and S11 was also tested. For all these assays, batches were prepared as described above, adding KNO_3_ (20 mM) as electron acceptor. OD was measured at 600 nm, and nitrate, nitrite, and sulfate were analyzed.

#### Aerobic versus anoxic SLES degradation

SLES cleavage and conversion to biomass and CO_2_ by strain S11 was compared at anoxic and oxic conditions, using 500 mg SLES L^−1^. Anoxic batch bottles were prepared as described above. For oxic conditions, bottles were prepared with air as gas phase. Nitrate, nitrite, sulfate, anionic surfactants, and DOC were analyzed at the beginning, after 1 day and 2 weeks of incubation. All batches were gently stirred (60 rpm; Innova 2300, New Brunswick Scientific, Edison, NJ) to avoid foaming.

#### SLES degradation by cocultures

The anoxic SLES degradation by strain S11 alone and by the consortium of strains S7, S8, and S11 was also compared after an incubation period of 3 weeks. Batch serum bottles were prepared as previously described, containing 250 mg L^−1^ of SLES and 10 mmol L^−1^ of KNO_3_. DOC measurements were performed at the beginning and at the end of the incubation.

#### SLES degradation by type strains

Type strains *Aeromonas hydrophila* DSM 30187^T^, *Pseudomonas stutzeri* CCUG 11256^T^, *P. nitroreducens* DSM 14399^T^, and *Comamonas denitrificans* DSM 17887^T^ were tested for SLES degradation coupled to nitrate reduction. *P. stutzeri* CCUG 11256^T^ was obtained from the Culture Collection of the University of Göteborg (Göteborg, Sweden). The other three cultures were purchased from the Deutsche Sammlung von Mikroorganismen und Zellkulturen (DSMZ, Germany). Batch serum bottles were prepared as previously described, containing 250 mg L^−1^ of SLES and 10 mmol L^−1^ of KNO_3_, and incubated for 2 weeks. Nitrate, nitrite, and sulfate were analyzed at the beginning and at the end of the incubation period.

### Analytical methods

OD at 600 nm was determined using a Hitachi U2000 UV/visible spectrophotometer (Hitachi, Tokyo, Japan). Nitrate, nitrite, and sulfate were analyzed by suppressor-mediated ion chromatography using a conductivity detector and an IonPac AS9-SC 4 × 50 mm column (Dionex, Sunnyvale, CA). The mobile phase was 1.8 mmol L^−1^ Na_2_CO_3_ and 1.7 mmol L^−1^ NaHCO_3_ at a flow rate of 1 mL min^−1^. Mannitol was added for stabilization of the samples, and sodium fluoride was used as internal standard. The analysis was conducted at 35 °C. Samples for DOC, anionic surfactant, and anion measurements were centrifuged and filtered using a membrane filter (0.22 μm) before analysis. Samples for DOC and anionic surfactant measurements were further acidified by adding 0.5 mL of H_2_SO_4_ (1 mol L^−1^). A TOC analyzer (TOC-VCSH, Shimadzu, Kyoto, Japan) was used for DOC measurements of the liquid samples. SLES concentration measurements were performed using anionic surfactants cell test kits (0.05–2 mg L^−1^ of methylene blue active substances (MBAS)) and a Spectroquant Multy photometer according to the manufacturer instructions (Merck, Darmstadt, Germany). A calibration curve was included to convert MBAS concentrations to mg SLES L^−1^.

## Results

### Bacterial diversity in enrichment cultures and isolation of bacteria

Five denitrifying enrichment cultures were obtained using SLES concentrations from 50 to 1000 mg L^−1^. At the eighth transfer, nitrate (about 8 mmol L^−1^) was completely reduced to nitrogen gases in the 250, 500, and 1000 mg SLES L^−1^ enrichments. In the 50 and 100 mg SLES L^−1^ enrichments about 1 and 3 mmol L^−1^ nitrate was reduced, respectively. Seven pure cultures were obtained from the 50, 250, and 1000 mg SLES L^−1^ enrichments (Table 1). All isolates were identified by comparison with their closest related described strains, with a minimum of 99% similarity based on their 16S rRNA gene sequence. Bacterial strains from the genera *Pseudomonas* and *Aeromonas* were obtained. Considering SLES degradation ability and 16S rRNA gene sequences similarity between the isolates (Table 1), three strains (*A. hydrophila* S7, *P. stutzeri* S8, and *P. nitroreducens* S11) were selected for further tests.

**Table 1:** Identification of obtained isolates and growth observations with SLES, under nitrate-reducing conditions

Strain code	Identification	Growth^a^	Enrichment^b^
S1	*Aeromonas hydrophila* (99% similar to *A. hydrophila* DSM 30187^T^)	+/−	50
S3	*A. hydrophila* (99% similar to *A. hydrophila* DSM 30187^T^)	+/−	250
S6	*Pseudomonas nitroreducens* (99% similar to *P. nitroreducens* DSM 14399^T^)	+/−	250
S7	*A. hydrophila* (99% similar to *A. hydrophila* DSM 30187^T^)	+/−	250
S8	*Pseudomonas stutzeri* (99% similar to *P. stutzeri* CCUG 11256^T^)	+	1000
S10	*P. stutzeri* (99% similar to *P. stutzeri* CCUG 11256^T^)	+	1000
S11	*P. nitroreducens* (99% similar to *P. nitroreducens* DSM 14399^T^)	+	1000

Based on 16S rRNA gene comparison (http://ncbi.nlm.nih.gov/blast), strains S1, S3, and S7 are 99% similar; strains S6 and S11 are 99% similar; strains S8 and S10 are 99% similar. All strains were also tested for SLES degradation with oxygen as electron acceptor; similar growth observations were obtained as presented in this table

^a^Growth observations were compared to controls without SLES; +/− weak but visible growth; + growth

^b^Original enrichment where the strain was isolated from; 50, 250, and 1000 correspond to the 50, 250, and 1000 mg SLES L^−1^ enrichments, respectively

**Fig. 2:**
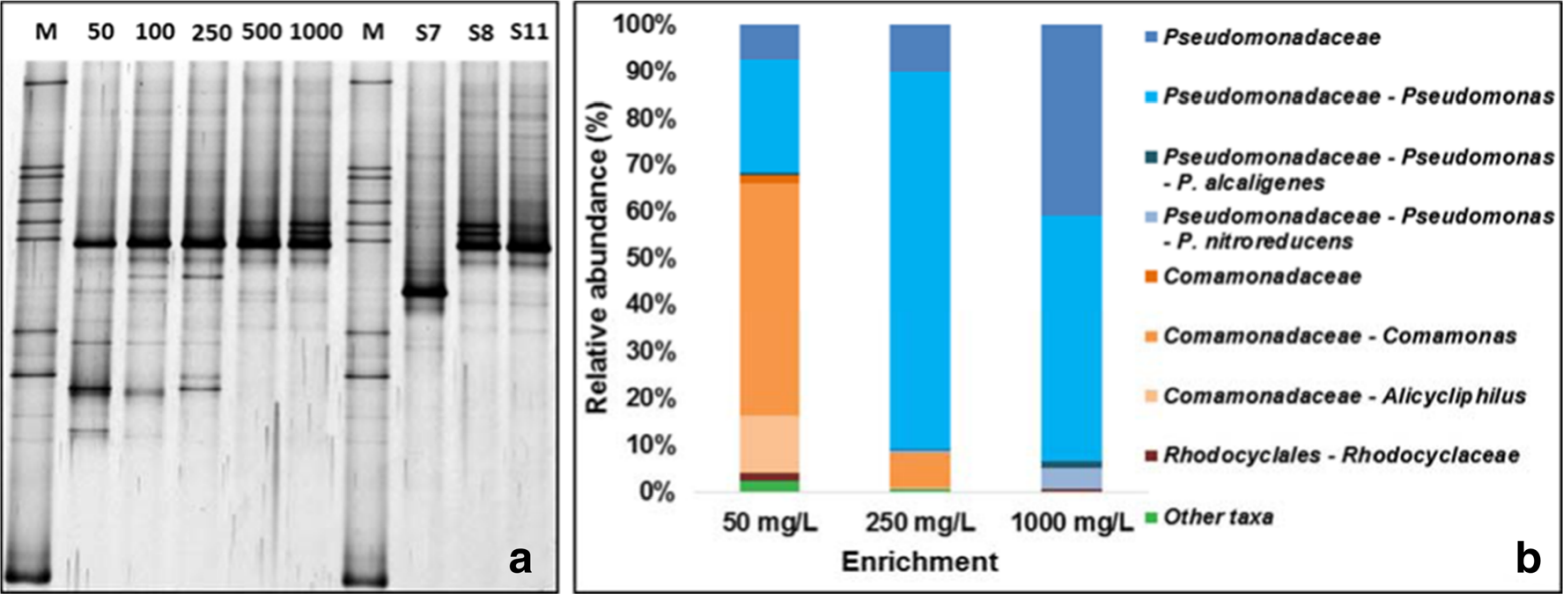
**a** DGGE analysis of bacterial 16S rRNA amplicons from enriched cultures and from selected isolates. Numbers from 50 to 1000 refer to SLES concentration (mg SLES L^−1^); *M* marker, *S7 Aeromonas hydrophila* strain S7, *S8 Pseudomonas stutzeri* strain S8, *S11 Pseudomonas nitroreducens* strain S11. **b** Relative abundance of taxa identified in the 50, 250, and 1000 mg SLES L^−1^ enrichments. Taxa with relative abundance ≤1% and with classification above the order level were included in *Other taxa*. Note: This taxonomical classification was obtained considering the complete classification of each OTU

The bacterial 16S rRNA amplicon profiles visualized by DGGE analysis of all enrichments and of the three isolates are shown in Fig. 2a. The number of bands in the enrichments decreased with increasing SLES concentration. The intense band present at the same migration position, in all the enrichment cultures, pointed to the abundance of *Pseudomonas*. This can be deduced from the migration of the amplified DNA of *P. stutzeri* strain S8 and *P. nitroreducens* strain S11 in the DGGE gels. The DGGE band profile of the 1000 mg L^−1^ enrichment is similar to the profile of *P. stutzeri* strain S8. Vague bands at the position corresponding to *A. hydrophila* strain S7 are visible in most lanes.

The relative abundance of the bacteria identified in the 50, 250, and 1000 mg SLES L^−1^ enrichments, using pyrosequencing analysis, is shown in Fig. 2b. Bacteria were classified to the family or genus level and in two cases to the species level. The Shannon and Chao1 indexes were calculated for the three enrichment samples. The Shannon index was 2.63, 1.83, and 2.07, for the 50, 250, and 1000 mg SLES L^−1^ enrichments, respectively. The Chao index was 49.4, 40.4, and 20.9, for the 50, 250, and 1000 mg SLES L^−1^ enrichments, respectively. All these results show that the structure of the bacterial community changed with SLES concentration. In the 50 mg SLES L^−1^ enrichment the genera *Comamonas* (50%), *Pseudomonas* (24%), and *Alicycliphilus* (12%) were present at higher relative abundance compared to the other enrichments. A large decrease in bacterial diversity was observed between 50 and 250 mg SLES L^−1^ enrichments. The decrease in the Shannon index reflects the decrease of the relative abundance of *Comamonas* and *Alycicliphilus* and the increase of the relative abundance of *Pseudomonas* and other bacteria from the *Pseudomonadaceae* family. *Pseudomonas alcaligenes* was identified in all enrichments at low relative abundance (0.5–1.5%). *P. nitroreducens* was identified in the 1000 mg SLES L^−1^ enrichment with 4% of relative abundance. Selected OTUs identified by QIIME as *Comamonas* and *Alicycliphilus* (50 mg L^−1^) were identified as *C. denitrificans* (99%) and *Alicycliphilus denitrificans* (99%) by BLASTN, respectively. *Pseudomonas* identified by QIIME in the 1000 mg SLES L^−1^ enrichment was identified as *P. stutzeri* (98%) by BLASTN.

As the ability for anaerobic SLES degradation is a remarkable property of our strains, *A. hydrophila* strain S7, *P. stutzeri* strain S8, and *P. nitroreducens* strain S11 are currently maintained in the culture collection of DSMZ at comparable conditions as described here.

### SLES degradation

Growth of *A. hydrophila* strain S7, *P. stutzeri* strain S8, and *P. nitroreducens* strain S11 with 500 mg SLES L^−1^ and nitrate was compared (Fig. S1). Final values are summarized in Table 2. From the three isolates, strain S7 grew poorly and reached a maximum OD value of 0.07 ± 0.00 after more than 6 days, with a doubling time of 32 h. Both strains S8 and S11 reached the maximum OD in less than 1 day, with a doubling time of 5 h. Compared with the other isolates, strain S11 reached the highest OD (0.25 ± 0.01), and cleaved and degraded more SLES (around 42 and 30%, respectively), while reducing nitrate to nitrogen gases, without accumulating nitrite (Table 2). Sulfate accumulation in the medium occurred simultaneously with bacterial growth and stabilized in the stationary phase for all three isolates (Fig. S1). In all cultures, more SLES was cleaved than degraded (Table 2).

**Table 2:** Conversion of SLES by strains S7, S8, and S11 under anoxic conditions (average values ± standard deviation)

Parameters	Strain S7	Strain S8	Strain S11
NO_3_^−^_red_ (mmol L^−1^)	11.1 ± 0.6	6.7 ± 0.5	5.5 ± 1.1
NO_2_^−^ (mmol L^−1^)	9.1 ± 0.5	4.6 ± 0.5	0.0 ± 0.0
SO_4_^2−^ (mmol L^−1^)	0.36 ± 0.02	0.31 ± 0.02	0.45 ± 0.05
OD_max_	0.07 ± 0.00	0.14 ± 0.01	0.25 ± 0.01
SLES_T0_ (mmol L^−1^)	1.28 ± 0.05	1.12 ± 0.02	1.46 ± 0.08
SLES_cleav_ (%)	33.1 ± 1.7	29.7 ± 2.2	41.6 ± 0.6
DOC_deg_ (%)	19.4 ± 1.2	24.8 ± 0.9	29.6 ± 1.8

*NO*_*3*_^*−*^_*red*_ nitrate reduced, *NO*_*2*_^*−*^ nitrite accumulated in the medium, *SO*_*4*_^*2−*^ sulfate accumulated in the medium, *OD*_*max*_ maximum OD obtained after 114, 15, and 19 h for strains S7, S8, and S11, respectively, *SLES*_*T0*_ estimated initial concentration of SLES, *SLES*_*cleav*_ SLES cleaved, *DOC*_*deg*_ SLES converted to biomass and CO_2_

### Effect of increased concentrations of surfactants

**Fig. 3:**
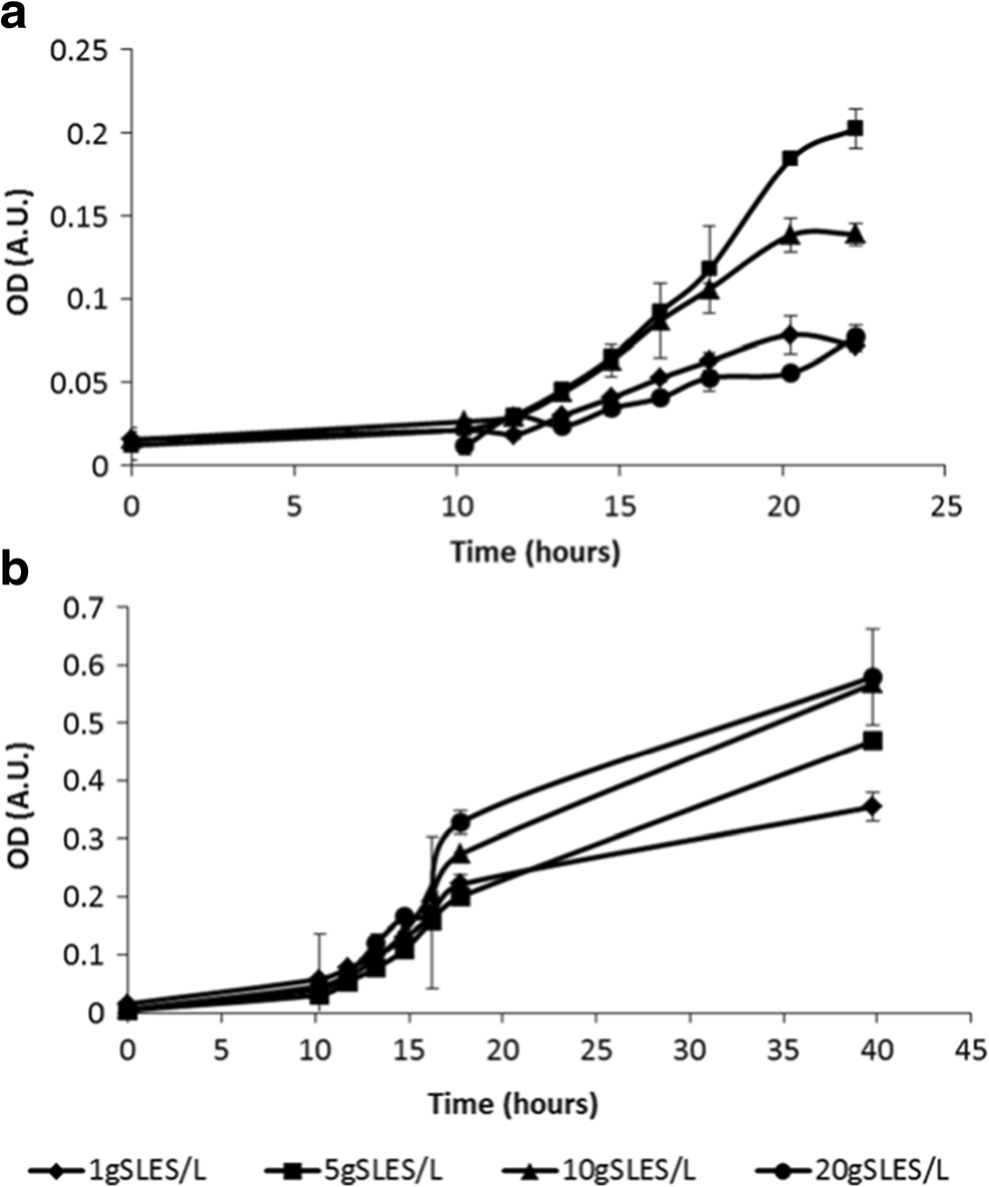
ODs increase until stationary growth of strains S8 (**a**) and S11 (**b**), with 1, 5, 10, and 20 g SLES L^−1^. *Symbols*: 1 g SLES L^−1^ (*diamonds*); 5 g SLES L^−1^ (*squares*); 10 g SLES L^−1^ (*triangles*); 20 g SLES L^−1^ (*circles*). Average values and standard deviation are presented

**Fig. 4:**
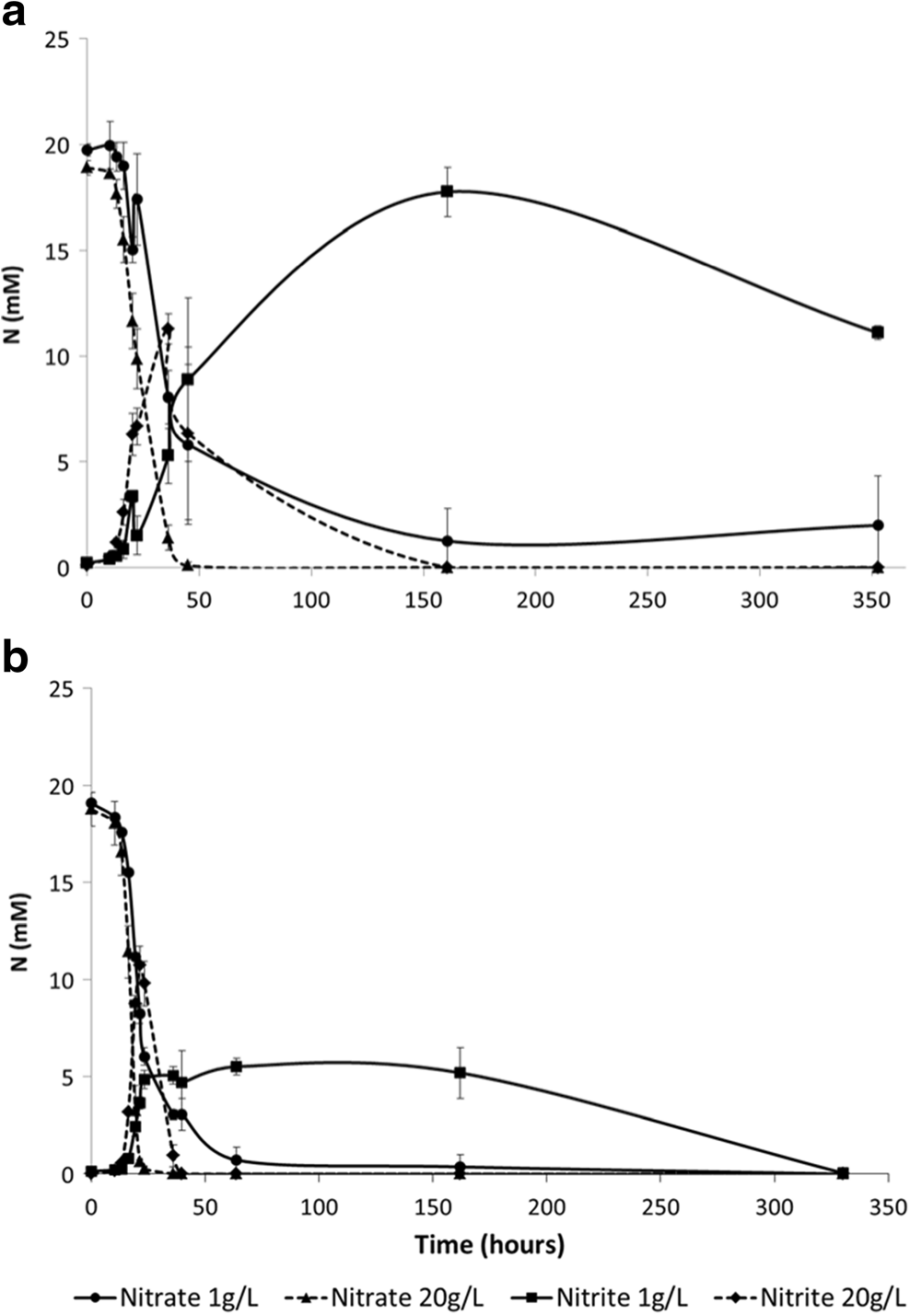
Nitrate reduction and nitrite accumulation during incubations of strains S8 (**a**) and S11 (**b**) with 1 and 20 g SLES L^−1^. *Symbols*: nitrate reduction with 1 g SLES L^−1^ (*circles*); nitrate reduction with 20 g SLES L^−1^ (*triangles*); nitrite accumulation with 1 g SLES L^−1^ (*squares*); nitrite accumulation with 20 g SLES L^−1^ (*diamonds*). Average values and standard deviation are presented

*P. stutzeri* strain S8 and *P. nitroreducens* strain S11 were compared for their ability to grow and reduce nitrate in the presence of higher SLES concentrations, between 1 and 20 g L^−1^ (Fig. 3 and Table 3). Both strains grew with all tested SLES concentrations. In all the assays, strain S11 reached a higher OD compared to strain S8. Maximum growth of strain S8 was achieved with 5 g SLES L^−1^, while strain S11 reached the highest OD with 10 and 20 g SLES L^−1^. Nitrate was completely removed with all SLES concentrations, by both strains. Nitrite accumulated in strain S8 culture only with 1 g SLES L^−1^. Results for nitrate reduction and nitrite accumulation with 1 g SLES L^−1^ and 20 g SLES L^−^1, for both strains, are shown in Fig. 4. Sulfate accumulated in the medium; maximum accumulation was obtained with 10 g SLES L^−1^, by both strains (Fig. S2). Strains S8 and S11 were also able to reduce nitrate (20 mmol L^−1^) when incubated with 40 g SLES L^−1^. In this assay, strain S8 reduced all added nitrate (20 mmol L^−1^) and accumulated about 20 mmol L^−1^ of nitrite, while strain S11 reduced all nitrate to nitrogen gases. When tested with the related anionic surfactant SDS, strain S8 grew less (OD ± standard deviation: 0.045 ± 0.023 and 0.128 ± 0.045, for 1 and 10 g SDS L^−1^, respectively) compared to strain S11 (OD ± standard deviation: 0.470 ± 0.059 and 0.512 ± 0.025, for 1 and 10 g SDS L^−1^, respectively), although both strains reduced all nitrate (20 mmol L^−1^) to nitrogen gases.

**Table 3:** Growth, nitrate reduction and sulfate accumulation of strains S8 and S11 with 1, 5, 10, and 20 g L^−1^ of SLES (average values ± standard deviation)

		SLES concentration			
Strains	Parameters	1 g L^−1^	5 g L^−1^	10 g L^−1^	20 g L^−1^
S8	NO_3_^−^_red_ (mmol L^−1^)	16.7 ± 2.5	18.0 ± 0.4	18.7 ± 0.4	18.9 ± 0.3
	NO_2_^−^ (mmol L^−1^)	10.9 ± 0.2	0.0 ± 0.0	0.0 ± 0.0	0.0 ± 0.0
	SO_4_^2−^ (mmol L^−1^)	1.2 ± 0.5	4.5 ± 0.1	7.2 ± 0.1	3.9 ± 0.3
	OD_max_	0.072 ± 0.004	0.203 ± 0.012	0.139 ± 0.007	0.072 ± 0.005
S11	NO_3_^−^_red_ (mmol L^−1^)	19.1 ± 0.1	19.3 ± 0.6	18.6 ± 0.3	18.8 ± 0.9
	NO_2_^−^ (mmol L^−1^)	0.0 ± 0.0	0.0 ± 0.0	0.0 ± 0.0	0.0 ± 0.0
	SO_4_^2−^ (mmol L^−1^)	1.3 ± 0.1	3.6 ± 0.3	6.5 ± 0.1	3.0 ± 0.2
	OD_max_	0.354 ± 0.024	0.468 ± 0.046	0.568 ± 0.65	0.580 ± 0.083

Initial NO_3_^−^ concentration was around 19 mM for all tests

*NO*_*3*_^*−*^_*red*_ nitrate reduced, *NO*_*2*_^*−*^ nitrite accumulated in the medium, *SO*_*4*_^*2−*^ sulfate accumulated in the medium, *OD*_*max*_ after 22 and 40 h for strains S8 and S11, respectively

### Aerobic versus anoxic SLES degradation

*P. nitroreducens* strain S11 was studied further by comparing SLES degradation with oxygen and nitrate (Table 4). After 2 weeks of incubation with oxygen, SLES was almost completely cleaved by strain S11 (99%), while in the incubation with nitrate SLES cleavage reached 66%. SLES conversion to biomass and CO_2_ was about 78 and 41% for the oxic and anoxic conditions, respectively. Sulfate release to the medium was compared to the concentration of SLES cleaved and SLES converted to biomass and CO_2_. Considering SLES molecular formula (Fig. 1), the amount of SLES converted to biomass and CO_2_ can be related to the amount of sulfate released. At the end of the incubation at oxic conditions, sulfate in the medium (0.70 mmol L^−1^) was close to the concentration of sulfate predicted from SLES conversion to biomass and CO_2_ (about 0.85 mmol L^−1^), but lower than the SLES cleaved (about 1.10 mmol L^−1^). For anoxic conditions, the situation was different since the sulfate accumulated (about 0.82 mmol L^−1^) was almost twice the sulfate predicted from SLES degradation (about 0.45 mmol L^−1^), but closer to the concentration of SLES cleaved (0.71 mmol L^−1^).

**Table 4:** SLES cleavage, SLES degradation, and sulfate accumulation by strain S11 at anoxic and oxic conditions, after 1 and 14 days of incubation (average values ± standard deviation)

	Time (days)	SO_4_^2−^ (mmol L^−1^)	SLES_cleav_ (mmol L^−1^)	SLES_cleav_ (%)	DOC_deg_ (%)
Anoxic	1	0.49 ± 0.01	0.42 ± 0.08	38.7 ± 4.8	28.6 ± 0.6
	14	0.82 ± 0.04	0.71 ± 0.09	65.6 ± 2.8	40.7 ± 5.2
Oxic	1	0.62 ± 0.03	0.80 ± 0.02	72.4 ± 2.3	56.4 ± 2.5
	14	0.70 ± 0.02	1.10 ± 0.02	99.6 ± 0.1	78.1 ± 0.8

Initial concentration of SLES was about 1.1 mmol L^−1^, for both anoxic and oxic assays

*SO*_*4*_^*2−*^ sulfate accumulated in the medium, *SLES*_*cleav*_ SLES cleaved, *DOC*_*deg*_ SLES converted to biomass and CO_2_

### SLES degradation by cocultures

SLES degradation by strain S11 alone and in a consortium with strain S7 and strain S8 was compared. After 3 weeks of incubation, SLES degradation by strain S11 alone was almost half of the value obtained by the consortium of the three bacteria (49.0 ± 1.6 and 85.9 ± 2.8%, respectively).

### SLES degradation by type strains

Based on 16S rRNA gene sequence analysis, *A. hydrophila* strain S7, *P. stutzeri* strain S8, and *P. nitroreducens* strain S11 are 99% similar to the respective type strains. These type strains were also tested for growth with SLES at anoxic conditions. *A. hydrophila* DSM 30187^T^ and *P. stutzeri* CCUG 11256^T^ did not grow, neither reduced nitrate using SLES as substrate. *P. nitroreducens* DSM 14399^T^ was able to grow with SLES and nitrate. Nitrate reduction resulted in nitrite and nitrogen gas formation (data not shown). *C. denitrificans* DSM 17887^T^, as a close relative of one of the dominant OTUs identified by BLASTN in the enriched cultures, was also tested for anoxic SLES degradation, but it could not use this surfactant as sole carbon and energy source.

## Discussion

This study reports for the first time the enrichment and isolation of SLES-degrading bacteria at anoxic conditions (nitrate-reducing), potentially involved in anoxic biodegradation of anionic surfactants in WWTPs. The importance of bacteria from the *Pseudomonas* genus in SLES conversion under denitrifying conditions was shown.

Different SLES concentrations were applied, which resulted in a selective pressure that leads to the reduction of bacterial diversity in the enrichments with high SLES concentration (Fig. 2, Chao1 and Shannon indexes). The growth of *Comamonas*, *Pseudomonas*, and *Alicycliphilus* was favored in the 50 mg SLES L^−1^ enrichment, while *Pseudomonas* were identified in all the five enrichments and became predominant at higher SLES concentration (Fig. 2). Since the first interaction locus of surfactants with bacteria is the membrane, a surfactant concentration near or above the CMC can solubilize bacterial cell membrane lipids and lead to cell lysis (Glover et al. 1999; Li and Chen 2009). Bacteria capable of SLES degradation may possess resistance mechanisms that help to counteract these potential toxic effects. In this work, *P. stutzeri* strain S8 and *P. nitroreducens* strain S11 reduced nitrate with SLES concentrations up to 40 g SLES L^−1^, showing a notable resistance to this surfactant.

Bacteria from the genus *Comamonas* have been associated with the aerobic degradation of sulfated and sulfonated surfactants (Matcham et al. 1977; Taranova et al. 2004; Weiss et al. 2012), but no growth was observed during the anoxic incubation of the type strain *C. denitrificans* DSM 17887^T^ with SLES. The ability of *Alicycliphilus* to degrade surfactants was also never shown. Therefore, it cannot be excluded that these two bacteria can use intermediary products resulting from SLES conversion, in the enrichments with lower SLES concentrations.

*A. hydrophila* strain S7, *P. stutzeri* strain S8, and *P. nitroreducens* strain S11 were isolated from the enrichments and are capable of SLES cleavage and degradation under denitrifying conditions (Table 2). Although bacteria from the *Aeromonas* and *Pseudomonas* genera are known to be involved in aerobic degradation of anionic surfactants (Asok and Jisha 2012; Chaturvedi and Kumar 2011; Jimenez et al. 1991; Sacco et al. 2006), the ability of *A. hydrophila*, *P. stutzeri*, and *P. nitroreducens* to use SLES as sole carbon and energy source coupled to nitrate or oxygen reduction was never shown before. In this work we verified that *A. hydrophila* (DSM 30187^T^) and *P. stutzeri* (CCUG 11256^T^) type strains cannot degrade SLES with nitrate as electron acceptor, which shows that SLES degradation might be a specific physiological ability of isolates S7 and S8. The type strain of *P. nitroreducens* (DSM 30187^T^) was able to grow with SLES and nitrate.

*P. nitroreducens* strain S11 was a better SLES degrader compared to the other two isolated strains. Strain S11 cleaved and converted a higher amount of SLES to biomass and CO_2_ compared to strains S7 and S8 (Table 2, Fig. S1) and grew to its highest OD value with 10 and 20 g SLES L^−1^, opposite to strain S8 (Table 3, Fig. 3).

Strain S11 did not accumulate nitrite, differently from strains S7 and S8 (Tables 2 and 3, Fig. S1). *A. hydrophila* is not described as a complete denitrifier; it was described to reduce nitrate only to nitrite (Knight and Blakemore 1998). This was also observed when *A. hydrophila* strain S7 was grown with acetate and nitrate (data not shown). On the other hand, *P. stutzeri* bacteria are known denitrifiers. Nitrite accumulated when *P. stutzeri* strain S8 was grown with 500 and 1000 mg SLES L^−1^ (Tables 2 and 3), but not when grown with 5, 10, and 20 g SLES L^−1^ (Table 3, Fig. 4). SLES is a commercial product that also contains other AES surfactants in its composition besides the main molecular structure described by the manufacturer (Fig. 1, *n* = 2 and R with 12 carbons). If only partial SLES degradation is achieved by strain S8, lower SLES concentrations represent less electron donor. The lack of electron donor can lead to the competition for electrons by nitrate and nitrite reductases, giving origin to nitrite accumulation (Almeida et al. 1995). This can explain nitrite accumulation by strain S8 in the presence of lower SLES concentrations. High concentration of nitrite was also measured when strain S8 was grown with 40 g SLES L^−1^, which may be due to a lower resistance of this strain to SLES negative effect, comparatively to strain S11.

Better growth with SLES was achieved by strain S11 compared to strain S8 (Tables 2 and 3, Fig. 3), and this was also the case when these strains were incubated with 1 and 10 g L^−1^ of SDS, another sulfated anionic surfactant. This might be related with differences in the physiology of the two *Pseudomonas* species. Taxonomically, *P. nitroreducens* has been placed in the *P. aeruginosa* group (Anzai et al. 2000). *P. aeruginosa* is a pathogenic bacterium resistant to biocides (Russell 1995) and several surfactant-degrading *P. aeruginosa* have been isolated (Swisher 1987). A greater resistance to surfactants might be shared among *P. aeruginosa* and close related *Pseudomonas* species.

Earlier studies were focused on the aerobic degradation of AES. Therefore, anoxic and aerobic SLES/AES degradation by a pure bacterial culture was never compared. Most of SLES was cleaved and converted to biomass and CO_2_ by strain S11 after 1 day of incubation, showing ability to perform fast aerobic and anoxic SLES degradation (Table 4). For each condition, the higher percentage of SLES cleaved relative to the percentage of SLES converted to biomass and CO_2_ indicates that some unknown intermediate compounds remained in the medium after SLES cleavage. Moreover, the relationship between sulfate accumulation, SLES cleavage, and conversion to biomass and CO_2_ was different between anoxic and oxic conditions (Table 4). This might be related to different mechanisms used for SLES cleavage in these two situations. The results obtained from the aerobic incubation support the occurrence of ether cleavage in this condition, associated with the release of sulfate only after SLES complete degradation (Fig. 1). In anoxic assays, SLES degradation through ester bond cleavage seems more significant, with most of the sulfate being released in the first cleavage step (Fig. 1). The production of sulfatases by *Pseudomonas* spp. able to degrade sulfated surfactants is well known (Gadler and Faber 2007). Besides, both strains S8 and S11 are able to use SDS, producing the sulfatases required for initial cleavage and further degradation. Although sulfatases can cleave AES surfactants through the ester bond and release sulfate, the intermediate compounds formed still contain ether bonds that must be cleaved to achieve complete mineralization of the surfactant (Hales et al. 1986). Since ether cleavage is not necessarily an oxygen-dependent mechanism (White et al. 1996), both ester and ether SLES cleavage might have occurred in anoxic conditions.

The complete degradation of SLES or an AES surfactant by one bacterium has not been observed so far, what suggests that the enzymes required for the complete degradation of these surfactants are present in a bacterial consortium. *A. hydrophila*, *P. stutzeri*, and probably also *C. denitrificans* and *A. denitrificans*, might be required for a complete degradation of SLES by *P. nitroreducens* strain S11. The high percentage (86%) of SLES degradation obtained with the consortium of strains S7, S8, and S11 supports this hypothesis.

This study shows SLES degradation at nitrate-reducing conditions. Three new nitrate-reducing strains able to degrade SLES were isolated. *Pseudomonas* were predominant at concentrations higher than the CMC value and strains S8 and S11 were able to grow with a SLES concentration 100 times higher than the CMC value (40 g SLES L^−1^), showing a remarkable resistance to this surfactant. *P. nitroreducens* strain S11 was the best SLES degrader, probably using combined ester and ether cleavage mechanisms. *Pseudomonas* are abundant in sewage sludge, and consequently, they will play an important role in the degradation of SLES/AES and other anionic surfactants disposed to WWTPs after domestic and/or industrial use. A fast anoxic degradation of high surfactants concentrations arriving to WWTPs prevents not only environmental problems but also disturbances of the activated sludge process.

## Electronic supplementary material

ESM 1 (DOCX 509 kb).
