# Supplementary material for: Sodium lauryl ether sulfate (SLES) degradation by nitrate-reducing bacteria
Source: Appl Microbiol Biotechnol. 2017 Mar 15;101(12):5163–73. doi: 10.1007/s00253-017-8212-x (PMC5486822; doi:10.1007/s00253-017-8212-x)
Supplement: Supplementary file 1 — (DOCX 509 kb). [file 253_2017_8212_MOESM1_ESM.docx]

***SUPPLEMENTARY MATERIAL***

**Sodium lauryl ether sulfate (SLES) degradation by nitrate-reducing bacteria**

**Applied Microbiology and Biotechnology**

Ana M. S. Paulo^a,b,c,#^, Rozelin Aydin^a,d^, Mauricio R. Dimitrov^a,*^, Harm Vreeling^a^, Ana J. Cavaleiro^c^, Pedro A. García-Encina^b^, Alfons J. M. Stams^a,c^ and Caroline M. Plugge^a^

^a^ Laboratory of Microbiology, Wageningen University, Stippeneng 4, 6708 WE Wageningen, The Netherlands

^b^ Department of Chemical Engineering & Environmental Technology, University of Valladolid, Calle Dr. Mergelina s/n, 47011 Valladolid, Spain

^c^ Centre of Biological Engineering, University of Minho, 4710-057 Braga, Portugal

^d^ Department of Bioengineering, Adana Science and Technology University, 01180 Seyhan/Adana, Turkey

Corresponding author e-mail: ana.paulo@ceb.uminho.pt


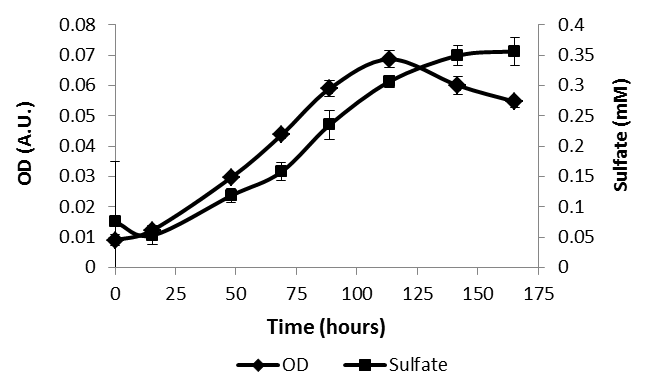

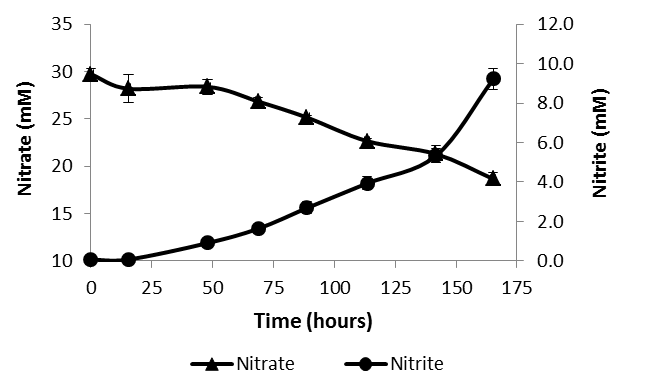

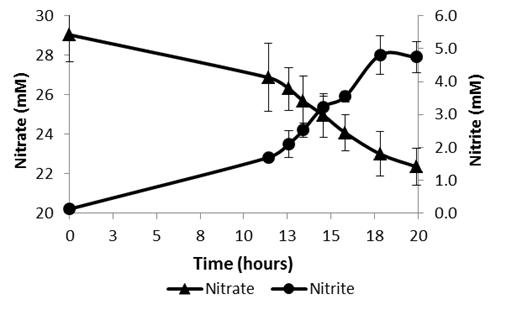

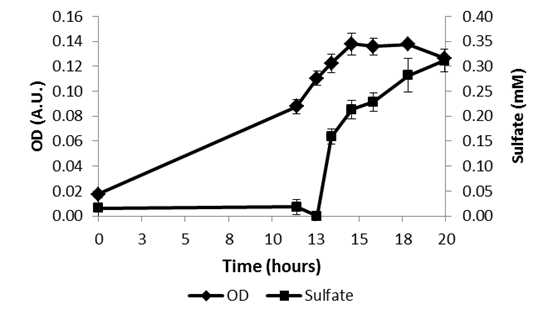

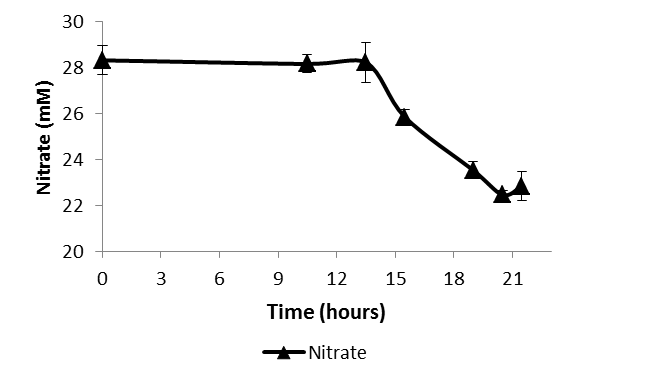

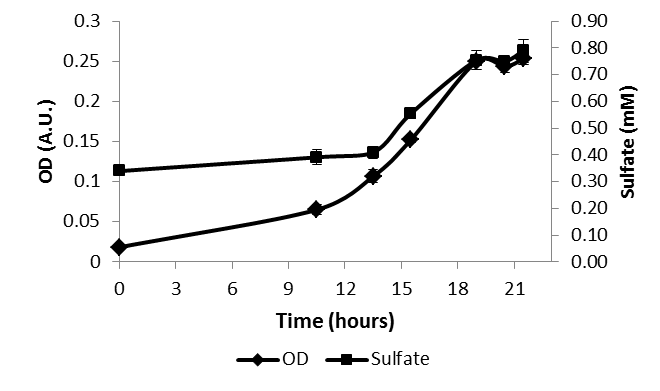


(a)

(b)

(c)

(d)

(e)

(f)

**Fig. S1** OD increase and sulfate accumulation during growth of strains S7 (a), S8 (b) and S11 (c); Nitrate reduction and nitrite accumulation during growth of strains S7 (d), S8 (e) and S11 (f); No accumulation of nitrite was detected during growth of strain S11. Symbols: (
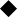
) - OD; (
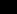
) – Sulfate; (▲) – Nitrate; (
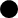
) – Nitrite. Average values and standard deviation are presented.


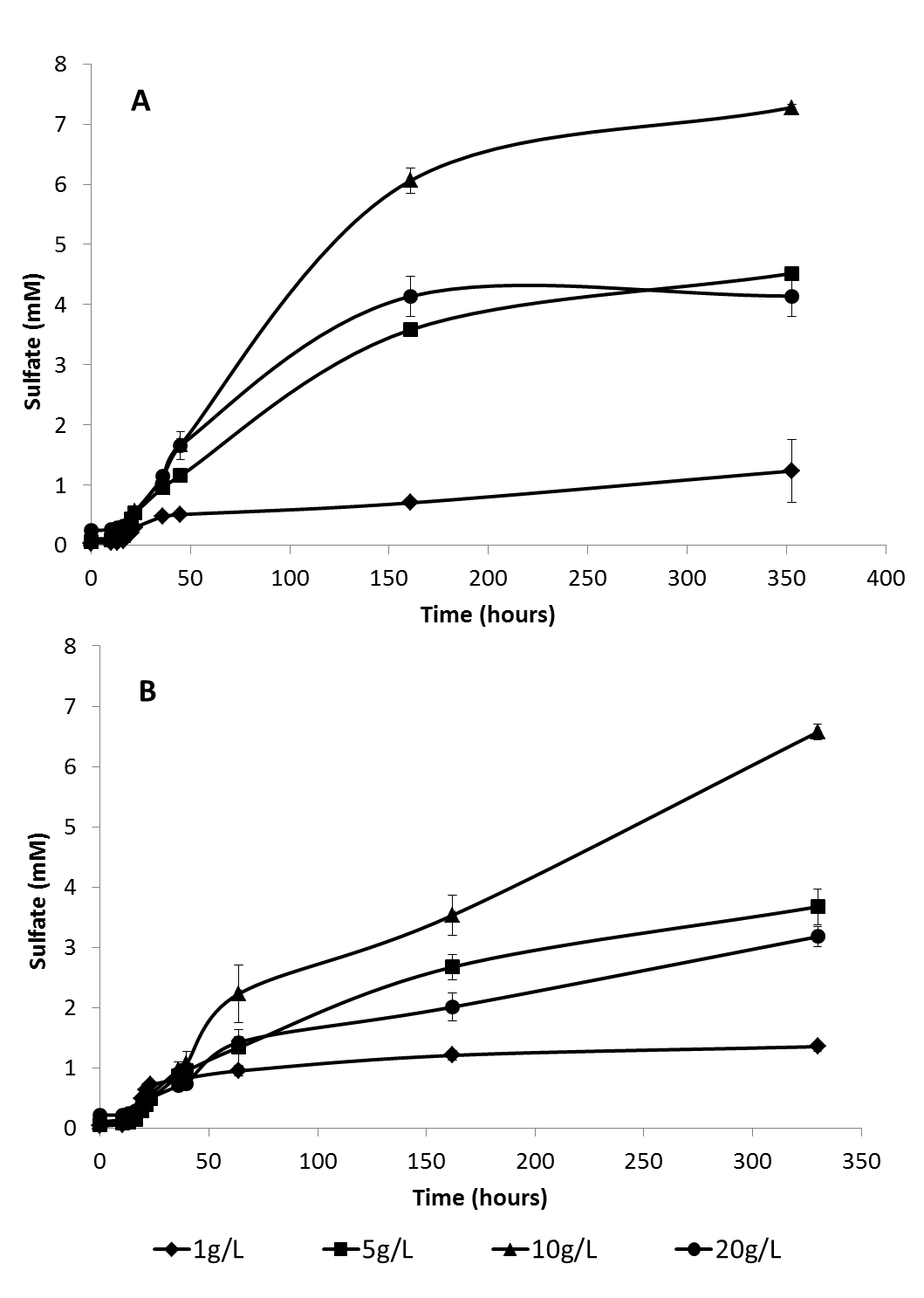


**Fig. S2** Sulfate accumulation during the assay with strains S8 (A) and S11 (B); Symbols: (
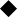
) – 1 g SLES L^-1^; (
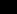
) – 5 g SLES L^-1^; (▲) – 10 g SLES L^-1^; (
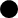
) – 20 g SLES L^-1^. Average values and standard deviation are presented.
